# Pyroglutamation of amyloid-βx-42 (Aβx-42) followed by Aβ1–40 deposition underlies plaque polymorphism in progressing Alzheimer's disease pathology

**DOI:** 10.1074/jbc.RA118.006604

**Published:** 2019-02-27

**Authors:** Wojciech Michno, Sofie Nyström, Patrick Wehrli, Tammaryn Lashley, Gunnar Brinkmalm, Laurent Guerard, Stina Syvänen, Dag Sehlin, Ibrahim Kaya, Dimitri Brinet, K. Peter R. Nilsson, Per Hammarström, Kaj Blennow, Henrik Zetterberg, Jörg Hanrieder

**Affiliations:** From the ‡ Department of Psychiatry and Neurochemistry, Sahlgrenska Academy at the University of Gothenburg, 43180 Mölndal, Sweden,; the § Department of Physics, Chemistry and Biology, Linköping University, 58183 Linköping, Sweden,; the ¶ Department of Neurodegenerative Disease, UCL Queen Square Institute of Neurology, University College London, London WC1N 3BG, United Kingdom,; the ‖ Department of Public Health and Caring Sciences, Uppsala University, 75236 Uppsala, Sweden,; the ** Clinical Neurochemistry Laboratory, Sahlgrenska University Hospital, 43180 Mölndal, Sweden,; the ‡‡ UK Dementia Research Institute at UCL, London WC1E 6BT, United Kingdom, and; the §§ Center for Cellular Imaging, Core Facilities, Sahlgrenska Academy at the University of Gothenburg, 41390 Gothenburg, Sweden

**Keywords:** amyloid-beta (Aβ), Alzheimer disease, mass spectrometry (MS), imaging, protein aggregation, neurodegeneration, pyroglutamation, plaque polymorphism, hyperspectral imaging, transgenic mice, pyroglutamic acid

## Abstract

Amyloid-β (Aβ) pathology in Alzheimer's disease (AD) is characterized by the formation of polymorphic deposits comprising diffuse and cored plaques. Because diffuse plaques are predominantly observed in cognitively unaffected, amyloid-positive (CU-AP) individuals, pathogenic conversion into cored plaques appears to be critical to AD pathogenesis. Herein, we identified the distinct Aβ species associated with amyloid polymorphism in brain tissue from individuals with sporadic AD (s-AD) and CU-AP. To this end, we interrogated Aβ polymorphism with amyloid conformation–sensitive dyes and a novel *in situ* MS paradigm for chemical characterization of hyperspectrally delineated plaque morphotypes. We found that maturation of diffuse into cored plaques correlated with increased Aβ1–40 deposition. Using spatial *in situ* delineation with imaging MS (IMS), we show that Aβ1–40 aggregates at the core structure of mature plaques, whereas Aβ1–42 localizes to diffuse amyloid aggregates. Moreover, we observed that diffuse plaques have increased pyroglutamated Aβx-42 levels in s-AD but not CU-AP, suggesting an AD pathology–related, hydrophobic functionalization of diffuse plaques facilitating Aβ1–40 deposition. Experiments in tgAPP_Swe_ mice verified that, similar to what has been observed in human brain pathology, diffuse deposits display higher levels of Aβ1–42 and that Aβ plaque maturation over time is associated with increases in Aβ1–40. Finally, we found that Aβ1–40 deposition is characteristic for cerebral amyloid angiopathy deposition and maturation in both humans and mice. These results indicate that N-terminal Aβx-42 pyroglutamation and Aβ1–40 deposition are critical events in priming and maturation of pathogenic Aβ from diffuse into cored plaques, underlying neurotoxic plaque development in AD.

## Introduction

The conspicuous phenotypic variability of AD^3^ remains poorly understood, which makes it challenging to establish a common molecular basis of AD pathology. AD heterogeneity was previously linked to molecular and morphological traits of individual β-amyloid (Aβ) deposits (1, 2). The formation of extracellular Aβ plaques has been identified as a major pathological hallmark of AD and a critical trigger of AD pathogenesis (3). According to the amyloid cascade hypothesis, it was suggested that the phenotypic heterogeneity of AD pathology is induced by polymorphic Aβ fibrils that precipitate as heterogeneous plaque pathology, including (formation of) diffuse plaques and cored, mature plaques (4–8).

Morphologic heterogeneity of Aβ plaques has been linked to the structural and chemical diversity of amyloid fibrils that consist of different Aβ peptide isoforms (9). These polymorphic fibrils are formed through structural transitions of different Aβ peptide isoforms during the aggregation process (1, 2).

Plaque polymorphism, attributed to differing fibrillary components, has been shown to correspond to distinct spectral emission upon luminescent conjugated oligothiophene (LCO)-based fluorescent amyloid staining (10–12). Specifically, plaque diversity, as delineated by differential amyloid dye staining, was previously attributed to distinct amyloid traits that were found to be specific for different familial forms of AD as well as in genetic mouse models of AD carrying the same mutations (13–15). On the histopathological level, this comprised varying patterns of both diffuse and cored Aβ plaque pathologies (16–19). Importantly, predominantly diffuse Aβ plaque pathology with almost no cored plaques has also been identified in cognitively unaffected amyloid-positive (CU-AP) individuals (20, 21). This suggests that both the differing Aβ plaque morphotypes and also molecular polymorphism at the Aβ fibril level and the associated Aβ peptide isoforms are of importance for explaining the heterogeneity of AD pathology. Whereas previous efforts have established that phenotypic heterogeneity of AD subtypes is reflected in morphological traits of individual plaque structures, associated biochemical characteristics, including Aβ peptide pattern, could not be delineated. We hypothesize that Aβ plaque polymorphism is associated with a plaque-specific Aβ peptide truncation pattern.

A major limitation in delineating amyloid pathology has been the lack of imaging techniques to concomitantly acquire chemical and structural information of individual Aβ aggregates. To test our hypothesis, we therefore developed a multimodal chemical imaging paradigm for delineating plaque polymorphism and the associated Aβ peptide signatures in post-mortem human brain from sporadic AD (s-AD) and CU-AP individuals as well as in a transgenic AD mouse model (tgAPP_SWE_) (22).

The results obtained here provide evidence for a relationship between Aβ peptide species ratio and Aβ plaque morphotypes (diffuse and cored), as indicated by conformational characteristics of Aβ plaques and the underlying peptide aggregates. Furthermore, as revealed by experiments in transgenic tgAPP_SWE_ mice, such structural transition of the fibrils underlying those Aβ plaques likely reflects plaque maturation.

## Results

### Hyperspectral imaging delineates structural characteristics of amyloid plaque polymorphism

Structural polymorphism of Aβ plaque pathology can be delineated in an unbiased way by using novel, fluorescent amyloid probes based on LCOs. These probes have different binding affinities to different amyloid structures as well as different electro-optic properties due to their flexible backbone, allowing these molecules to adopt different backbone structures. Different LCOs can therefore be delineated using hyperspectral detection in fluorescent microscopy (23).

To understand how Aβ plaque polymorphism is related to distinct Aβ peptide content, we investigated structural and chemical characteristics of individual plaques in post-mortem human brain tissue from the temporal cortex of sporadic AD in the dementia stage and CU-AP cases (Table S1) as well as in transgenic AD mice (tgAPP_SWE_).

**Figure 1.**
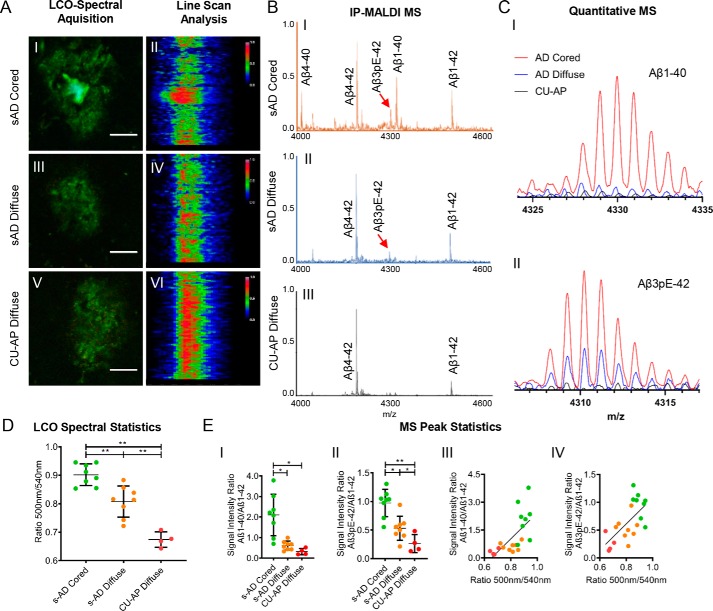
**Spectral and mass spectrometric analysis of amyloid deposits in s-AD and CU-AP patients.** *A*, LCO microscopy; double staining was performed with q-FTAA and h-FTAA. Cross-sectional emission profile of cored (*A.I* and *A.II*) and diffuse plaques in s-AD (*A.III* and *A.IV*) compared with diffuse plaques in CU-AP patients (*A.V* and *A.VI*). Hyperspectrally classified plaques were then excised with laser microdissection and extracted with formic acid followed by immunoprecipitation (IP) and MALDI MS. *B*, MALDI MS; MALDI mass spectra for immunoprecipitated plaque extracts from laser microdissected cored plaques (*B.I*) and diffuse plaques (*B.II*) in s-AD patients and diffuse plaques in CU-AP patients (*B.III*). *C*, *zoomed* MALDI MS traces; overlay of average mass spectra for Aβ1–40 (*C.I*) and AβpE3–42 (*C.II*), indicating higher levels of Aβ1–40 in cored plaques (*red*) compared with diffuse plaques and increased levels of AβpE3–42 in s-AD (*red* and *blue*) as compared with CU-AP (*black*). *D*, statistics on hyperspectral emission ratio values (500 nm/540 nm) corresponding to the degree of q- and h-FTAA content. *E*, statistics on Aβ1–40 and AβpE3–42 MS signal between plaque groups (*E.I* and *E.II*) and correlation with 500-nm/540-nm emission ratio for Aβ1–40/Aβ1–42 (*R*^2^ = 0.43, *p* < 0.005) (*E.III*); and AβpE3–42/Aβ1–42 (*R*^2^ = 0.41, *p* < 0.005) (*E.IV*). The number of patients was as follows: *n* = 8 (s-AD) and *n* = 4 (CU-AP). 200–250 cored- and 200–250 diffuse plaques for s-AD and 200–250 diffuse plaques for CU-AP were collected from five consecutive temporal cortical sections per patient. *Scale bar* (*A*), 25 μm. *Error bars* (*D*), S.D. Significance is shown as follows: *, *p* < 0.05; **, *p* < 0.005.

To delineate spectral characteristics of Aβ polymorphism in human and mouse brain tissue, we used a double-stain strategy with two LCO-based amyloid probes, tetra- and heptameric formyl-thiophene acetic acids (q- and h-FTAA) (Fig. 1*A* and Fig. S1 (*A* and *B*)). This hyperspectral imaging paradigm was used for unbiased annotation of structurally distinct plaque morphotypes (*i.e.* cored and diffuse plaques) (Fig. S1, *B* and *C*). The aim was then to characterize the corresponding Aβ peptide profile by isolating these plaques using laser microdissection with pressure catapulting (LMPC) followed by immunoprecipitation and mass spectrometric analysis (IP-MS, Fig. S1*C*).

Using this chemical imaging paradigm allowed us to annotate mature, Congo red (CR)-positive, Aβ fibrils as well as immature fibrillary intermediates of Aβ aggregation that are not detectable by thioflavin S or Congo red as described previously (Fig. 1*A* and Figs. S1*B* and S2) (10).

In the s-AD cases, we identified two major groups of Aβ plaque morphotypes, cored and diffuse, based on their morphology as well as their characteristic hyperspectral emission profiles that reflect differential LCO binding. Here, cored plaques exhibited a heterogeneous emission profile with red emission at 540 nm at the periphery, indicating h-FTAA binding, along with a characteristic blue shift at the center region, corresponding to preferential q-FTAA binding (Fig. 1, *A.I* and *A.II*). In contrast, morphologically diffuse plaques in s-AD showed a homogeneous emission profile at 540 nm across the entire plaque area, indicating h-FTAA binding (Fig. 1, *A.III* and *A.IV*) (10, 11, 13).

In contrast to s-AD pathology, brain tissue from CU-AP cases showed almost exclusively diffuse plaque morphotypes that exhibited emission profiles similar to the diffuse plaques observed in s-AD cases (Fig. 1, *A.V* and *A.VI*).

Given the spectral difference that we observed for the different plaque morphotypes, we sought to quantify differential LCO-binding in all plaques. For this, we calculated the mean emission ratio at 500 nm/540 nm, corresponding to the ratio of bound q-FTAA/h-FTAA (10). The results showed that q-FTAA staining in cored plaques was 14% higher than in diffuse plaques in s-AD and 25% higher than in diffuse plaques in CU-AP (Fig. 1*D*).

Complementary, co-staining experiments of the LCOs with thioflavin S and Congo red as well as birefringence spectroscopy of CR show that q-FTAA–positive aggregates as observed in cored plaques are more fibrillar in structure as compared with h-FTAA–positive diffuse amyloid structures (Fig. S2*A*).

Thus, our results suggest that diffuse plaques in s-AD and CU-AP are structurally similar and consist of immature, fibrillary Aβ aggregation intermediates, whereas cored plaques are characterized by formation of mature, q-FTAA- and CR-positive Aβ fibrils.

### The Aβ1–40/Aβ1–42 ratio is associated with heterogeneous plaque morphology

To characterize the Aβ composition pattern of these different plaque types, we isolated hyperspectrally annotated Aβ plaque morphotypes using laser microdissection (Fig. S1, *C.II*). We extracted and selectively enriched Aβ species from the collected plaques, using a two-step immunoprecipitation approach (Fig. S1, *C.III*). The individual Aβ species in the precipitate were then characterized using MS (Fig. S1, *C.IV*), resulting in chemically specific MS peak data (Fig. 1, *B* and *C*), allowing for relative quantification of individual Aβ species in the plaque extracts (Fig. 1*E*). Further, the detected mass signals were verified by high-resolution MS and MS/MS (Figs. S3–S5)

Our results showed that the Aβ1–40/Aβ1–42 ratio was 3.5-fold higher in cored plaques than in diffuse plaques in the s-AD group (Fig. 1*B*, *C.I* and *E.I*) and 7-fold higher than in the diffuse plaques found in the CU-AP group (Fig. 1*B*, *C.I* and *E.I*). We observed a similar pattern for Aβ4–40 and Aβ4–42, the N-terminally truncated isoforms of Aβ1–40 and Aβ1–42 (Fig. S6*A*). Here, Aβ4–42 was the most dominant peak in the MS spectrum of all plaque types (Fig. 1*B*). The Aβ4–40/Aβ4–42 ratio was 4-fold higher in cored plaques than in diffuse plaques in s-AD and 20-fold higher than in diffuse plaques present in CU-AP (Fig. S6*A*). These results suggest that Aβ1–40 and Aβ4–40 are associated with formation of cored plaques and more mature Aβ fibrils in heterogeneous plaque pathology observed in AD dementia brains, whereas, as mentioned previously, CU-AP brains contained almost exclusively diffuse plaques that consist mostly of Aβ1–42 and Aβ4–42, respectively (Fig. 1*B*.*III*).

Given this pronounced increase of Aβ1–40 and Aβ4–40 in cored plaques, we investigated whether the relative amounts of these Aβ species correlated with the hyperspectral LCO signals. The results showed that Aβ1–40/Aβ1–42 correlated significantly with 500 nm/540 nm (*R*^2^ = 0.43, *p* < 0.005; Fig. 1*E.III*). This indicates a positive association of Aβ1–40 with q-FTAA fluorescence and of Aβ1–42 with h-FTAA fluorescence. The correlation results for the ratio of the corresponding N-terminally truncated species, Aβ4–40/Aβ4–42, showed the same positive associations with 500 nm/540 nm (*R*^2^ = 0.36, *p* < 0.01; Fig. S6*B*). This suggests that Aβx-40 species are associated with cored plaque areas, whereas Aβx-42 peptides correlate with diffuse Aβ structures.

### Pyroglutamate modification of Aβx-42 is increased in amyloid plaques in AD

Whereas Aβ1–40 deposition was found to be the key parameter associated with cored plaques, the results further show that the main chemical difference between diffuse plaques found in AD and CU-AP includes a significant increase in N-terminal pyroglutamate (pE) species of Aβ1–42 (AβpEx-42).

Our results showed that the AβpE3–42/Aβ1–42 ratio was 2-fold higher in diffuse plaques in s-AD than in diffuse plaques in CU-AP and 3 times higher in cored plaques in s-AD than in the diffuse plaques found in the CU-AP group (Fig. 1, *C.II* and *E.II*). We observed a similar pattern for AβpE11–42, where the AβpE11–42/Aβ1–42 ratio was 2 times higher in both cored and diffuse plaques in s-AD as compared with diffuse plaques present in CU-AP (Fig. S7, *A.I*). Similarly to the Aβ1–40/Aβ1–42 ratio data, we asked whether the relative amounts of the AβpE species correlated with the hyperspectral LCO signals. The results showed that both AβpE3–42/Aβ1–42 (*R*^2^ = 0.41, *p* < 0.005; Fig. 1*E.IV*) and AβpE11–42/Aβ1–42 (*R*^2^ = 0.32, *p* < 0.01; Fig. S7*A.II*) correlated significantly with 500 nm/540 nm. These results suggest that pyroglutamate modification of Aβ1–42 is associated with Alzheimer-specific Aβ pathology.

### Amyloid β1–40 localizes to the center of cored plaques, whereas Aβx-42 species localize to diffuse aggregates

**Figure 2.**
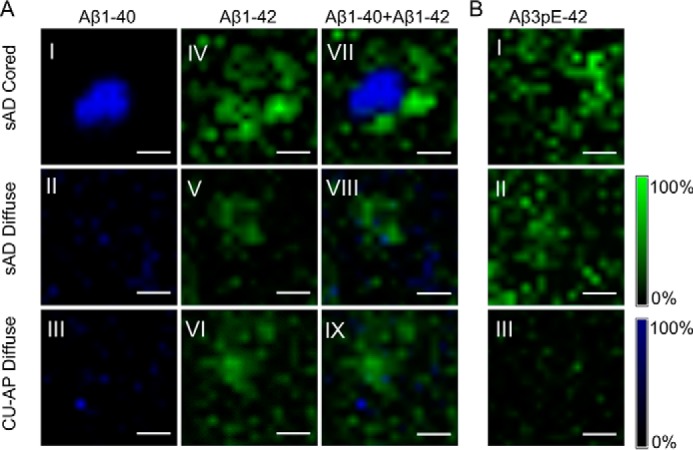
**MALDI Imaging for delineating intraplaque Aβ heterogeneity.** *A*, MALDI imaging MS (IMS); single-ion images of individual plaques from MALDI IMS analysis revealed a prominent localization of Aβ1–40 to the center of cored plaques in s-AD (*A.I*), whereas the Aβ1–42 signal localized to the periphery of cored plaques in s-AD tissue (*A.IV*; see also the image overlay (*A.VII*)). Diffuse plaques in both s-AD (*A.II* and *A.V*) and CU-AP (*A.III* and *A.VI*) showed low Aβ1–40 signal, but a strong Aβ1–42 signal, that was homogeneous across these plaques as highlighted in the *overlay images* (*A.VIII* and *A.IX*). *B*, MALDI IMS analysis further revealed localization of AβpE3–42 to the periphery of cored plaques in s-AD (*B.I*) as well as diffuse plaques in s-AD (*B.II*), whereas only a very low signal was present for these peptides in diffuse plaques in CU-AP (*B.III*). MALDI IMS was performed on consecutive sections to the sections used for LCO imaging and LMPC. The number of patients was as follows: *n* = 8 (s-AD) and *n* = 4 (CU-AP). *Scale bar* (*A* and *B*), 25 μm. *Intensity scales* indicate the maximum peak intensities of MALDI single-ion signal.

Whereas the LMPC-IP–based *in situ* MS method of hyperspectrally differentiated plaque morphotypes provided chemical signatures associated with Aβ polymorphism, no spatially resolved Aβ peptide identification data can be obtained on the single-plaque level. We thus performed MALDI imaging MS (IMS) on s-AD and CU-AP tissue to resolve the localization of distinct Aβ peptides within single plaques and to delineate how these correlate with the LCO staining results (Fig. 2*A*). Here, we observed that the Aβ1–40 signal was primarily localized to the center of cored plaques in s-AD brain tissue but was not detected in diffuse plaques in s-AD and CU-AP as visualized in the single ion images (Fig. 2, *A.I–A.III*). In contrast, Aβ1–42 distributed to the periphery of cored plaques (Fig. 2, *A*.*IV* and *A.VII*). Further, Aβ1–42 was strongly localized to diffuse plaques in both s-AD (Fig. 2, *A.V* and *A.VIII*) and CU-AP (Fig. 2, *A.VI* and *A.IX*). These results are well in line with our LMPC-IP-MS data (Fig. 1) and further verify that indeed Aβ1–40 (and Aβ4–40; Fig. S6*C*) is associated with mature Aβ fibrils and q-FTAA staining, respectively, whereas Aβ1–42 (and the more dominant Aβ4–42 signal (Fig. S6*D*)) is associated with diffuse, monofilamentous, protofibrillar Aβ assemblies that are found in diffuse plaques both in s-AD and CU-AP. Further, in line with the full-length Aβ1–42, the corresponding pE species, AβpE3–42 and AβpE11–42, showed localization to diffuse areas of cored plaques (Fig. 2*B*.*I* and Fig. S7*C.I*) as well as diffuse plaques in s-AD (Fig. 2*B.II* and Fig. S7*C.II*) and CU-AP (Fig. 2*B.III* and Fig. S7*C.III*).

### Chemical characteristics of amyloid plaque polymorphism in humans are equivalent to tgAPP_SWE_ mouse model

**Figure 3.**
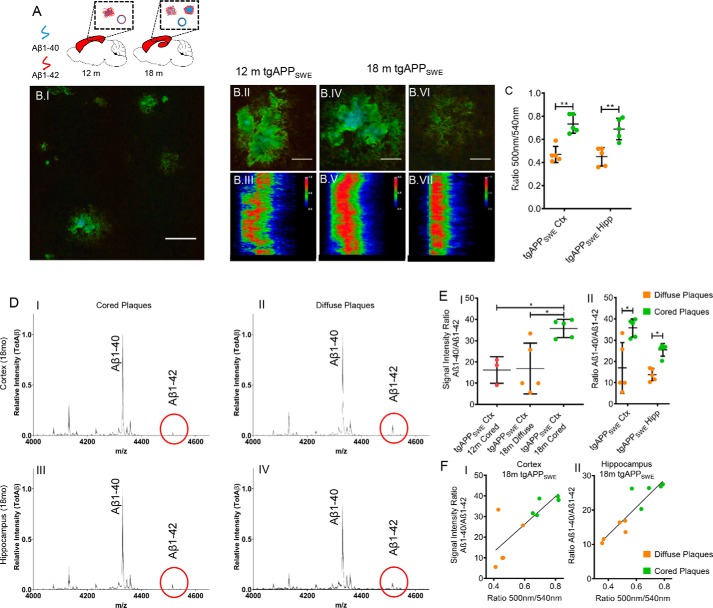
**Aβ deposits in aging tgAPP_SWE_ mice.** *A*, Aβ plaque pathology and vascular Aβ deposition were investigated in 12-month-old (*n* = 3) and 18-month-old male tgAPP_SWE_ mice (*n* = 5) in the cortex and hippocampus. *B.I*, wide field, hyperspectral fluorescence microscopy of cortex in 18-month-old tgAPP_SWE_ mice shows heterogeneous plaque pathology. *B.II*, hyperspectral microscopy of cortical plaques in 12-month-old tgAPP_SWE_ mice shows small compact plaques with the presence of a core (*B.II*), as further reflected in an intermediate cross-sectional emission profile (*B.III*). Shown is LCO fluorescence imaging of cortical Aβ plaque pathology in 18-month-old tgAPP_SWE_ mice, with zoom (*B.IV*) and cross-sectional emission profile of a cortical cored (*B.V*) and diffuse Aβ deposit (*B.VI* and *B.VII*). *C*, average 500-nm/540-nm ratio for distinct plaque subgroups in two brain regions of 18-month-old mice reveals a similar pattern between diffuse and cored deposits in both regions, resembling that of s-AD patients. Hyperspectrally classified plaques were then excised with laser microdissection and extracted with formic acid followed by immunoprecipitation and MALDI MS (IP-MALDI MS). *D*, corresponding MALDI MS spectra of different plaque types show relative Aβ peptide intensities in cored (*D.I* and *D.III*) and diffuse (*D.II* and *D.IV*) plaques in the cortex (*ctx*, *D.I* and *D.II*) and hippocampus (*hipp*, *D.III* and *D.IV*) of 18-month-old tgAPP_SWE_ mice (*n* = 5). The *red circles* highlight the Aβ1–42 peak, which is relatively higher in the diffuse deposits (*D.II* and *D.IV*), and that this trend is more prominent in cortical plaques (*D.I* and *D.III*). *E*, statistical analysis reveals that small, cored plaques in 12-month-old tgAPP_SWE_ mice display a Aβ1–40/Aβ1–42 ratio similar to that of diffuse plaques at 18 months. In contrast, the Aβ1–40/Aβ1–42 ratio was 2-fold higher in cored plaques in 18-month-old mice as compared with both diffuse plaque in 18-month-old mice and small, cored plaques in 12-month-old mice (*E.I*). Similarly, for the 18-month-old animals, the Aβ1–40/Aβ1–42 signal ratio was consistently 2-fold increased in cored plaques as compared with diffuse plaques in both the cortex and hippocampus (*E.II*). Correlation of the Aβ1–40/Aβ1–42 ratio with the 500-nm/540-nm emission ratio in the cortex (*F.I*, *R*^2^ = 0.64, *p* < 0.005) and hippocampus (*F.II*, *R*^2^ = 0.82, *p* < 0.005) is shown. The number of animals was as follows: *n* = 5 (18-month-old) and *n* = 3 (12-month-old). 15–20 cored and 15–20 diffuse plaques were collected from only cortex (12 months) and from both cortex and hippocampus (18 months) from five sagittal sections per animal. *Scale bar* (*B*), 75 μm; *zoom*, 25 μm. *Error bars* (*C* and *E*), S.D. Significance is indicated as follows: *, *p* < 0.05; **, *p* < 0.005.

Our hyperspectral imaging results obtained for plaque morphotypes in s-AD and CU-AP are in line with previous observations in transgenic models with Aβ pathology (10, 23, 24). To determine whether cored and diffuse plaque–specific spectral properties are reflected in a general shift in Aβ peptide ratio over time, we performed LMPC and IP-MS on LCO-delineated plaque morphotypes in 12- and 18-month-old tgAPP_SWE_ mice that displayed heterogeneous plaque pathology, including cored, diffuse plaques and cerebral amyloid angiopathy (CAA) (Fig. 3*A* and *B.I*) (25).

In 12-month-old mice, we observed deposition of small compact plaques that primarily localized to the cortex, whereas almost no plaque formation was observed in the hippocampus (Fig. 3, *A* and *B.II*). This is in line with previous findings in different transgenic mouse models carrying the Swedish double mutation of APP. These studies have reported an initial formation of smaller cored Aβ deposits at 10–12 months, followed by rapid and exponential growth of both cored and a few diffuse plaques, until full-blown plaque pathology is reached at 18 months (25–27). Our double-LCO staining results obtained from 12-month-old mice showed that these early Aβ plaques displayed a pronounced core structure (Fig. 3*B.II*). The emission profile across the center of these early small, compact plaques showed, however, a more heterogeneous blue shift (Fig. 3*B.III*), as compared with the spectral data observed for cored plaques in s-AD.

In 18-month-old mice, we observed Aβ plaque pathology with heterogeneous morphology and LCO-annotated optical properties in both the cortex and hippocampus. Here, we detected two major subpopulations of plaque morphotypes in both the cortex and hippocampus, including cored plaques and diffuse plaques, as observed for s-AD (Fig. 3, *B.IV–B.VII*). At this age, the majority of plaques did exhibit a Congo red–positive, core structure observed in bright field and birefringence microscopy (Fig. S2, *B* and *C*) (25) together with a pronounced q-FTAA staining in the center (Fig. 3, *B.IV* and *B.V*). In contrast, diffuse plaques showed a homogeneous emission profile at 540 nm across the plaque area, corresponding to h-FTAA (Fig. 3, *B.VI* and *B.VII*). Further, the emission wavelength ratio, 500 nm/540 nm (q-FTAA/h-FTAA) (10), was 60% higher in cored deposits as compared with diffuse ones in both the cortex and hippocampus (Fig. 3*C*).

We then determined the Aβ peptide profiles of all plaque types in all mice at both ages, using laser microdissection and IP-MS for relative quantification of the individual Aβ species based on their MS traces (Fig. 3*D*). Our results showed that there was no difference in Aβ1–40/Aβ1–42 ratio between cored plaques in 12-month-old mice and diffuse plaques in 18-month-old mice (Fig. 3*E.I*), In contrast, the Aβ1–40/Aβ1–42 ratio in cored plaques in older mice was 2 times higher than in cored plaques in 12-month-old mice (Fig. 3*E.I*).

In 18-month-old animals, the Aβ1–40/Aβ1–42 ratio in cored plaques was also 2 times higher as compared with diffuse plaques, which was consistent in both the cortex and the hippocampus (Fig. 3, *D* and *E.II*). Similar to the human data, we found that the spectral emission ratio, 500 nm/540 nm, correlated significantly with the Aβ1–40/Aβ1–42 peptide ratio in both the cortex (Fig. 3*F.I*; *R*^2^ = 0.64, *p* < 0.005) and the hippocampus (Fig. 3*F.II*; *R*^2^ = 0.82, *p* < 0.005). This indicates that, in accordance with the plaque characteristics observed in human Aβ pathology, q-FTAA binding correlates with Aβ1–40 levels and that Aβ1–40 is associated with formation of cored plaques.

**Figure 4.**
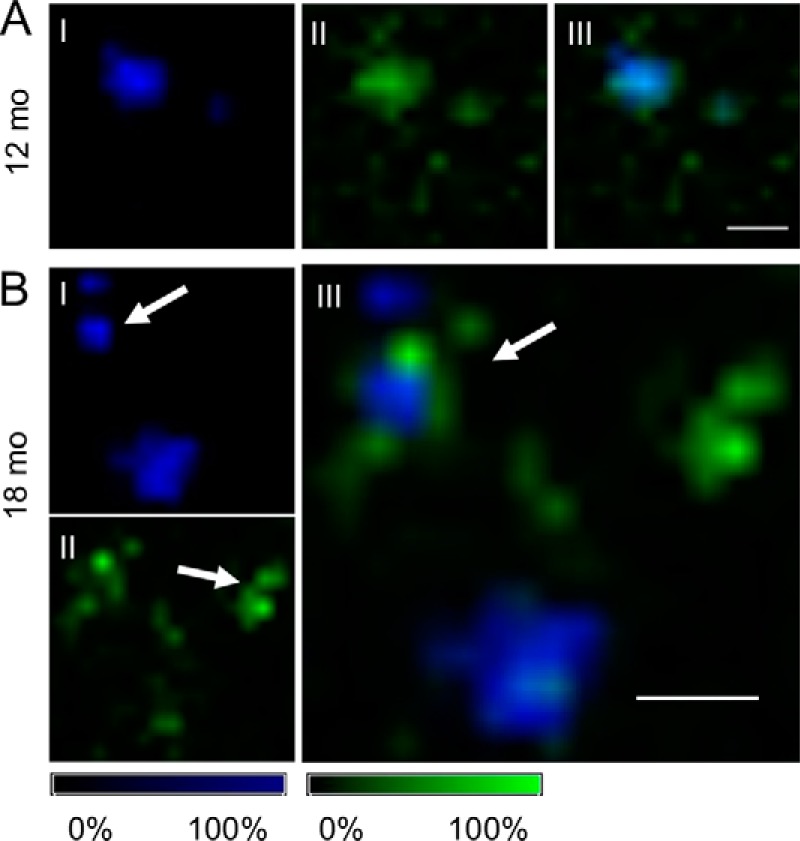
**MALDI imaging of Aβ deposits in aging tgAPP_SWE_ mice.** *A*, for 12-month-old mice, MALDI imaging MS (IMS) analysis revealed small compact plaques with high levels of Aβ1–40 (*A.I*) and moderate localization of Aβ1–42 to the periphery (*A.II*) while still displaying a high degree of (*A.III*) colocalization. *B*, in 18-month-old tgAPP_SWE_ mice, MALDI IMS reveals that Aβ1–40 predominantly localizes to core structures of cored plaques (*arrow*, *B.I*). In contrast, Aβ1–42 localized primarily to diffuse plaques (*arrow*, *B.II*) and diffuse radial structures of cored deposits, as seen in the *overlay image* (*arrow*, *B.III*). The number of animals was as follows: *n* = 5 (18-month-old) and *n* = 3 (12-month-old). MALDI IMS was performed on consecutive sections to the sections used for LCO imaging and LMPC. *Scale bar* (*A* and *B*), 75 μm. *Intensity scales* indicate maximum peak intensities of the MALDI single-ion signal.

To confirm the localization of Aβ1–40 to cored plaque structures, we further verified these results for LCO-outlined plaques using MALDI IMS on adjacent tissue sections (Fig. 4, *A* and *B*). The results showed localization of Aβ1–40 to core structures for small cored plaques in 12-month-old mice, whereas Aβ1–42 displayed a more heterogeneous localization, as illustrated in the individual ion images (Fig. 4, *A.I–A.III*).

For 18-month-old tgAPP_SWE_ mice, our MALDI IMS experiments showed peptide localization patterns similar to the findings observed in human tissue. Here, Aβ1–40 localized predominantly to the central core structures of cored deposits (Fig. 4*B.I*). In contrast, Aβ1–42 localized primarily to diffuse plaques and diffuse peripheral structures of cored deposits (Fig. 4, *B.II* and *B.III*).

This suggested that the LCO spectral pattern and the associated increase in Aβ1–40/Aβ1–42 peptide ratios are comparable between cored plaques observed in human AD as well as in the transgenic mouse model of AD. Moreover, these results show an age-associated change of spectral characteristics toward q-FTAA emission at 500 nm along with an increase in Aβ1–40/Aβ1–42 ratio. Overall, this suggests that plaque maturation associated with AD pathogenesis is associated with conformational rearrangement from diffuse to cored deposits (10) and that this process is characterized by an interplay of Aβ1–40 and Aβ1–42 during incorporation into maturing fibrils (28).

### CAA follows age-associated patterns observed for cored plaque

CAA is another characteristic in AD amyloid pathology and is suggested to be associated with the presence of cored amyloid deposits (29). Similarly, CAA is observed in both 12- and 18-month-old transgenic mice harboring the Swedish APP mutation (30–32). To investigate the chemical link between plaque polymorphism and vascular amyloidosis, we examined the LCO spectral characteristics and the associated Aβ peptide signature of CAA deposits in human s-AD and in tgAPP_SWE_ mice.

**Figure 5.**
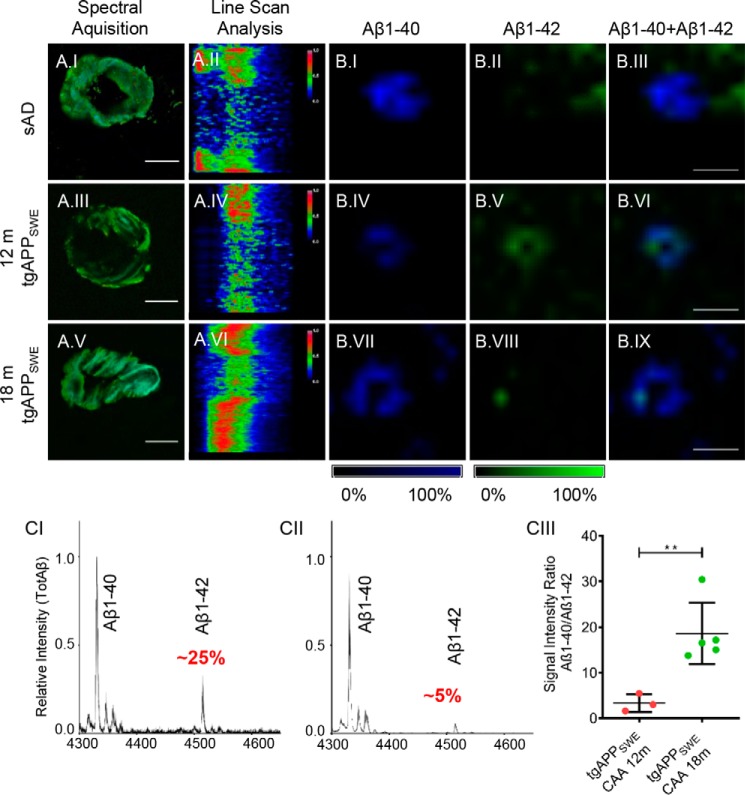
**Parenchymal CAA in s-AD patients and tgAPP_SWE_ mice.** Hyperspectral image and cross-sectional emission profile of CAA from s-AD tissue showed dominant q-FTAA emission (*A.I* and *A.II*). For 12-month-old (*A.III* and *A.IV*) and 18-month-old (*A.V* and *A.VI*) tgAPP_SWE_ mice, an age-dependent shift toward q-FTAA emission was observed. MALDI imaging MS (IMS) showed a strong Aβ1–40 signal (*B.I*) with low Aβ1–42 levels (*B.II* and *B.III*) in s-AD brain. In younger tgAPP_SWE_ mice, MALDI IMS verified that both Aβ1–40 (*B.IV*) and Aβ1–42 (*B.V*), are present in CAA, showing clear signal colocalization (*B.VI*). In older mice, CAA consisted mainly of Aβ1–40 (*B.VII*), with a weak Aβ1–42 signal (*B.VIII*) with colocalization (*B.IX*). The number of CAAs analyzed per patient was ∼50, and the number of CAAs analyzed per animal was 15–25. MALDI IMS was performed on consecutive sections to the sections used for LCO imaging and LMPC. Scale bar (*A*), 25 μm; (*B*), 75 μm. Intensity scales indicate maximum peak intensities of the MALDI single-ion signal. *C*, IP-MS mass spectra of parenchymal CAA in 12-month-old (*n* = 3) (*C.I*) and 18-month-old tgAPP_SWE_ mice (*n* = 5) (*C.II*). The relative amount of Aβ1–42 in relation to the Aβ1–40 peptide differed significantly, and comparative statistics of average ratios reveal a major, significant increase in relative amounts of Aβ1–40 in older mice (18 months) (*C.III*). 15–25 CAAs were collected from five sagittal sections per animal. *Error bars* (*C.III*), S.D. Significance is indicated as follows: **, *p* < 0.005.

Our results showed that CAA in s-AD patients showed a strong q-FTAA–positive, blue emission across the vessel wall of individual CAA deposits (Fig. 5, *A.I* and *A.II*). IP-MS of laser microdissected CAA from s-AD tissue showed a dominant signal for Aβ1–40 and Aβ4–40, whereas no Aβ1–42 was detected (data not shown). This was verified with MALDI IMS of individual CAA in human s-AD brain tissue, where Aβ1–40 was found to localize within the rim of CAA deposits (Fig. 5*B.I*). In contrast, no Aβ1–42 was found to localize to CAA deposits (Fig. 5, *B.II* and *B.III*).

In transgenic mice, CAA formation was observed in the cortex of 12-month-old mice and in the cortex and hippocampus in older animals at 18 months (Fig. 5, *A.III–A.VI*). Spectral analysis of LCO-stained brain tissue showed different cross-sectional emission profiles with increasing age. In 12-month-old mice, vascular amyloid deposits showed strong h-FTAA emission across their surface area (Fig. 5, *A.III* and *A.IV*). In 18-month-old mice, we observed a blue shift at the center of the vessel wall in these CAA structures, indicating a more mature Aβ aggregation state (Fig. 5, *A.V* and *A.VI*). IP-MS of laser-microdissected CAA from transgenic mice showed that the Aβ1–40/Aβ1–42 ratio was 5 times increased in CAA in 18-month-old animals as compared with CAA in 12-month-old mice (Fig. 5*C*).

To further verify the change in Aβ1–40/Aβ1–42 ratio, we performed MALDI IMS on CAA in tgAPP_SWE_ mouse brain. For 12-month-old mice, our IMS results show that both Aβ1–40 (Fig. 5*B.IV*) and Aβ1–42 (Fig. 5*B.V*) localize to CAA pathology (Fig. 5*B.VI*). In 18-month-old mice, we observed a strong localization of Aβ1–40 to CAA (Fig. 5*B.VII*), whereas the signal for Aβ1–42 showed only a weak localization to CAA (Fig. 5, *B.VIII* and *B.IX*). Taken together, the data obtained for plaque pathology in transgenic mice suggest that increased Aβx-40 deposition plays a crucial role in maturation of both vascular amyloidosis and extracellular amyloid plaques and core formation, respectively.

## Discussion

In this study, we investigated whether structural polymorphism of Aβ plaque morphotypes is associated with distinct Aβ chemistry. Our results show that cored plaques in s-AD are characterized by deposition of Aβ1–40, whereas diffuse plaques in both s-AD and CU-AP are characterized by deposition of Aβ1–42. Further, our data show that diffuse plaques in s-AD show increased levels of pyroglutamate-modified N-terminally truncated Aβ1–42 species (N-pyro-E-Aβ; AβpE3–42, AβpE11–42) as compared with diffuse plaques in CU-AP. Imaging MS identified a specific Aβ1–40 localization to the center of cored plaques, suggesting that Aβ1–40 is associated with mature amyloid structures and dense fibrils, respectively, within cored plaques in s-AD. In contrast, diffuse areas of cored deposits as well as diffuse plaques in both s-AD and CU-AP were largely composed of Aβ1–42. The corresponding pyro-E peptides AβpE3–42 and AβpE11–42 localized to diffuse structures as well.

Because plaques in CU-AP show primarily a diffuse morphology, these results suggest that full-length Aβ1–42, while being indicative of general amyloidosis, is not the primary neurochemical trait associated with Aβ pathogenicity and toxicity in AD.

These findings appear to stand in contrast to the current perception that Aβ1–42 is the most relevant Aβ species associated with AD pathogenesis as suggested by CSF biomarker findings, where decreased Aβ1–42 levels, but not Aβ1–40, point toward brain wide accumulation of Aβ1–42 (33–35).

Plaque pathology in CU-AP with diffuse Aβ deposits could therefore represent prodromal AD pathology that, given enough time, would progress toward formation of mature, cored amyloid plaques, as observed here and also previously reported for tgAPP_SWE_ (23) as well as APP23 and APP/PS1 mice (10). Our data on both human and mouse samples suggest that this maturation and core formation involve deposition of Aβ1–40 at the core.

Indeed, previous investigations on *in situ* Aβ quantification showed 20-fold higher levels of fibrillar Aβ1–40 and only 2-fold higher Aβ1–42 levels in brain tissue from AD patients, as compared with CU-AP patients (36, 37). Further, in immunohistochemistry-based studies, Aβ1–40 was suggested to be associated with cored plaque formation in s-AD along with predominant Aβ1–42 staining of diffuse plaques both in AD and CU-AP (38). Whereas pronounced CAA formation, characterized by predominant deposition of Aβ1–40, was shown to result in decreased CSF levels of Aβ1–40 in patients with severe CAA (39), no such results have been reported for AD-associated Aβ plaque pathology.

One could therefore speculate that the effect of this plaque-specific Aβ1–40 deposition is difficult to detect in CSF. Presumably, this is due to the general high abundance of Aβ1–40 in the brain, where the change in equilibrium of deposited and soluble Aβ1–40 as a consequence of plaque maturation (and Aβ1–40 deposition) is too minor to be reflected in the periphery.

An increase of Aβ1–42 in the brain, as indicated by decreased CSF levels, points to a general increased plaque load irrespective of plaque morphology and can be explained with Aβ1–42 being spherically accumulated in all plaques, including cored plaques, and thereby accounts for a significantly larger part of the plaque volume. Indeed, by comparing relative values, an increase in Aβ1–40/Aβ1–42 ratio seems to originate from increased Aβ1–40. Because Aβ1–40 is confined to the core structures that are smaller in volume relative to the total plaque volume, the amount may be underestimated by histological, antibody-based staining techniques. This is also consistent with Western blotting–based results reported on laser-microdissected plaques in s-AD, CU-AP, and tgAPP/PS2 mice, which showed that cored and diffuse plaques were found to contain predominantly Aβ1–42, whereas the Aβ1–40/Aβ1–42 ratio was higher in cored plaques as compared with diffuse plaques owing to a higher content of Aβ1–40 (40).

In line with this, our observations in tgAPP_SWE_ mice show an increased q-FTAA–staining pattern and Aβ1–40/Aβ1–42 ratio in cored plaques compared with diffuse plaques, which was demonstrated with LCO/LMPC and IP-MS as well as with imaging MS. These data are supported by previous, immunohistochemistry-based studies on plaque polymorphism in transgenic mice that demonstrated a prominent Aβx-40 immunoreactivity within plaque cores, whereas Aβx-42 was found to stain mostly the radial periphery of cored plaques as well as diffuse deposits (25, 27, 38, 41). The chemical and spectroscopic properties of diffuse parts of cored plaques as well as diffuse plaques in s-AD and diffuse plaques in CU-AP were consistent with respect to h-FTAA emission and Aβ1–42 content.

Given previous data on LCO-delineated plaque maturation in transgenic mice (10) and cross-seeded amyloidosis (42) and the here identified Aβ correlates, this suggests that diffuse plaques are precursors of cored plaques and that this maturation is associated with AD pathogenesis. This plaque maturation process is characterized by increased q-FTAA binding, and the corresponding chemical correlate is Aβ1–40 that accumulates within the core region of mature plaques upon nucleation.

This is further supported by our results from tgAPP_SWE_ mice, where we followed Aβ plaque pathology over time. Whereas the general sample size was not large, these data showed clear trends and statistically significant changes in chemical plaque pathology that were tantamount to the findings in human tissue. In detail, early compact plaques observed in 12-month-old mice show higher relative amounts of Aβ1–42 and h-FTAA staining as compared with cored plaques in 18-month-old animals. Chemically, the early compact plaques at 12 months were similar to diffuse plaques observed in older mice that also contain relatively higher amounts of Aβ1–42 as compared with cored plaques. This suggests again that an increase in Aβ1–40/Aβ1–42 ratio is associated with plaque maturation of diffuse plaques into cored plaques via recruitment and deposition of Aβ1–40. Based on our observations, a possible pathological mechanism of plaque formation suggests initial seeding of extracellular Aβ aggregation through accumulation of soluble Aβ1–42 that is predominantly secreted during rising amyloid (43). This is followed by nucleation and maturation upon recruitment of Aβ1–40, which is in line with previous observations in tgAPP_SWE_ mice (44).

Along that line, a prominent role of Aβ1–42 for initial plaque deposition has been suggested previously based on data in human AD brain (26) and transgenic mice (16) as well as for seeded Aβ pathology in different transgenic mice, including tgAPP/PS1 and tgAPP23 (42). Aβ1–42 has been shown to rapidly form oligomers and subsequently fibrils, as compared with other C-terminally truncated Aβ species (45). In contrast, independent mechanisms for cored plaque formation have been suggested based on experiments in different transgenics, where cored plaques are also observed in younger mice (31, 46, 47). This is in line with our observations for younger mice, where only small compact/cored plaques were observed. However, it is still under debate whether this is a consequence of massive APP overexpression and Aβ production leading to rapid plaque formation and nucleation in neocortical areas, which might not be representative for how Aβ pathology is initiated in human AD.

Together with the data on C-terminal Aβ species, our observations on increased pyroglutamate-modified N-terminally truncated Aβ42 (N-pyro-E-Aβ; AβpE3–42, AβpE11–42) in diffuse plaques in AD but not in CU-AP further suggest a prominent role of Aβ1–42 functionalization in seeding Aβ pathology in AD. Indeed, N-pyro-E-Aβ42 truncation has previously been identified to be prominent in brain extracts (37) and senile plaques in AD following initial Aβ1–42 aggregation (38, 48). Interestingly, AβpE3–42 has been suggested to be the dominating Aβ species in senile and diffuse plaques in AD, Downs syndrome, and CU-AP (38, 49, 50). In contrast, our data clearly show that the dominating species in all plaques is Aβ4–42 and that this truncation is not differing in between plaque types and disease state and is therefore rather a nonspecific metabolite of Aβ1–42. One explanation for this discrepancy is that all previous data were based on detection *in situ* or in brain extracts using an antibody toward Aβ pE3–42 that could be cross-reactive for Aβ4–42, something that has not been studied in these publications. Nevertheless, N-pyro-E-Aβ42 species have mechanistically been implicated in AD pathogenesis by accelerating Aβ aggregation kinetics because N-pyro-E-Aβ are more hydrophobic than the full-length species and are more potent for self- and co-aggregation of less hydrophobic Aβ species, including Aβ1–40 (51–54). Therefore, higher levels of AβpE3–42 and AβpE11–42 in cored and diffuse plaques in AD, but not in diffuse plaques in CU-AP, likely reflect an important role of N-pyro-E-Aβ42 in seeding Aβ aggregation and early stages of plaque formation. This process likely involves hydrophobic priming that eventually leads to deposition of less hydrophobic species, including Aβ1–40, that remain otherwise in solution.

Overall, these data indicate that Aβ1–42 and N-pyro-E-Aβ42 are relevant species in seeding pathology and that diffuse plaques represent an early stage of Aβ deposits that mature into cored plaques and that this process involves the recruitment of more hydrophilic Aβ1–40 species over time. Here aggregation and functionalization of Aβ1–42 via N-terminal pyroglutamation are critical for seeding Aβ pathology in AD, whereas Aβ1–40 was shown to be associated with mature amyloid fibril formation (55). Further, Aβ1–40 was demonstrated to be significantly less potent for seeding amyloid fibril formation as compared with Aβ1–42 (45, 56).

This notion is further supported by our observations for cerebrovascular amyloid pathology. Here, a strong localization of Aβ1–40 peptide along with dominating q-FTAA binding was demonstrated for CAA in s-AD as well as in tgAPP_SWE_. Further, in mice, similar to plaques, predominant Aβ1–40 deposition in CAA was found to increase with age. This suggests that CAA maturation is characterized by increased Aβ1–40 deposition.

This is in line with previous data, where development of CAA pathology has been shown to be associated with increased AD-associated mutations that result in increased secretion of total Aβ, such as due to the Swedish mutation in tgAPP_SWE_ mice (32, 57).

Further, Aβ1–42 as well as N-terminal Aβ truncations that are both prone to aggregation have previously been shown to readily deposit as fibrillary diffuse plaques while having no relevance in already seeded CAA or plaque nucleation (38, 58). Similar to our findings, these previous studies suggest that with progressing pathology, Aβ species less prone to aggregation, dominated by Aβ1–40, do deposit on the preseeded aggregation sites, both in amyloid plaques (leading to core formation) and in the vasculature (resulting in aggravated CAA pathology).

Importantly, the age-associated blue shift observed in CAA, caused by q-FTAA binding, along with increased Aβ1–40 deposition, indicates higher-order aggregation represented by denser fibrillary structures, such as bundled multifilamentous fibrils (12). These denser fibril structures might be associated with other physiological consequences, including stroke and hemorrhages. Indeed, CAA is associated with vascular Aβ clearance (59), and severe CAA pathology with frequent and spontaneous cerebral and lobar hemorrhages was described for both humans and transgenic AD mice (60–64). Given that hemorrhages occur due to decreasing flexibility in the endothelium of blood vessels (65), this suggests that differences in CAA-associated hemorrhage between different AD mutations are a consequence of higher rigidity of Aβ1–40–containing, mature fibrils. Indeed, Aβ1–40 fibrils were shown to be over 50 times less elastic than the Aβ1–42 fibrils (66), and this has been attributed to different β-sheet organization within each fibrillary layer of mature Aβ fibrils (66).

In summary, we identified that Aβ plaque polymorphism is associated with distinct Aβ peptide patterns. Specifically, we found that Aβ1–40 and not Aβ1–42 is the dominating species in mature senile plaques with cored morphology that have been implicated in AD pathogenesis. Further, this plaque maturation was found to be associated with increased levels of Aβ3pE-42, which could indicate a hydrophobic priming of diffuse plaque morphotypes in AD through pyroglutamate modification of N-terminally truncated Aβ42.

A limitation of our study is the relatively small number of patients analyzed. These cross-sectional data provide initial molecular insight into heterogeneous plaque pathology on a chemical scale, not previously possible, and are largely verified by the longitudinal mouse data. However, there is a strong motivation in using the here-described technologies for expanded follow-up studies both for longitudinal human studies and mechanistic studies in mice.

Taken together, our data suggest that diffuse deposits are immature precursors of cored plaques and that pyroglutamation of N-terminal Aβx-42 and Aβ1–40 deposition are potentially critical events in priming and maturation of pathogenic Aβ from diffuse into cored plaques. These processes could underlie development of neurotoxic plaque pathology in AD and could hence provide a mechanistic target for potential intervention.

## Experimental procedures

### Patient samples

Fresh brain tissue samples were obtained from temporal cortex of eight clinically and pathologically diagnosed sporadic AD cases (s-AD, AD1–AD8) and four nondemented CU-AP cases (CU-AP1–CU-AP4) (Table S1). All cases were obtained through the brain donation program of the Queen Square Brain Bank for Neurological Disorders, Department of Movement Disorders, UCL Institute of Neurology. The standard diagnostic criteria were used for the neuropathological diagnosis of AD (67–69). The demographic data for all cases are shown in Table S1. Ethical approval for the study was obtained from the Local Research Ethics Committee of the National Hospital for Neurology and Neurosurgery as well as the Institutional Review Board at the University of Gothenburg (Gothenburg, 04/16/2015; DNr 012-15). All studies abide by the Declaration of Helsinki principles.

### AD mouse model

Fresh brain tissue samples were obtained from 12-month-old (*n* = 3) and 18-month-old (*n* = 5) male transgenic AD mice (tgAPP_Swe_). Animals were reared *ad libitum* at an animal facility at Uppsala University under a 12-h/12-h light cycle (22). The animals were anesthetized with isoflurane and sacrificed by decapitation. The brains were dissected quickly with less than a 3-min post-mortem delay and frozen on dry ice. All animal procedures were approved by the ethical committee at Uppsala University (Uppsala, Sweden) (DNr C17/14) and performed in compliance with national and local animal care guidelines as well as in accordance with the principles of the Declaration of Helsinki.

### LCO staining

Two previously validated LCO fluorophores, q-FTAA and h-FTAA, were used for the staining of the fresh-frozen tissue (10, 13). Fresh-frozen human and mouse brain tissue was cut into 12-μm-thick sections on a cryostat microtome (Leica CM 1520, Leica Biosystems, Nussloch, Germany) at −18 °C, and consecutive sections were collected on 0.17 PEN membrane slides (Zeiss/P.A.L.M., Microlaser Technologies, Bernsried, Germany) and stored at −80 °C. Prior to staining, the sections were thawed in a desiccator and fixed at −20 °C for 10 min using 95% ethanol and double-stained with q-FTAA and h-FTAA (2.4 μm q-FTAA and 0.77 μm h-FTAA in PBS) similar to a protocol described previously (10, 13). Sections were incubated for 30 min at room temperature in the dark, rinsed with milliQ water, and finally dried through desiccation.

### Congo red and LCO co-staining

Congo red staining was performed on fresh frozen tissue sections (12 μm) that were fixed in 99% ethanol and rehydrated through 10-min dips in 70% ethanol, distilled H_2_O, and PBS, pH 7.3. Congo red staining of amyloid was performed as described previously, with few modifications (71). In short, tissue sections were first stained with Mayers hematoxylin for 1 min and destained in tap water and deionized water. Tissues were equilibrated in alkaline 80% EtOH for 20 min followed by Congo red staining solution for 20 min for mouse tissue and 2 h for human tissue. The Congo red staining solution was prepared by freshly filtered 0.2% (w/v) Congo red in alkaline 80% ethanol with 1% NaCl. Destaining was performed in deionized water and PBS, pH 7.3. Sections were mounted using transparent Dako fluorescence mounting medium.

Hyperspectral imaging of LCO-stained tissue sections was performed using a Leica DM6000 B fluorescence microscope (Leica, Wetzlar, Germany) equipped with a SpectraCube module (Applied Spectral Imaging, Migdal Ha-Emek, Israel). Imaging of Congo red–stained tissue sections was performed using a Nikon Eclipse 50i microscope with open, semi-crossed, and crossed polarizers, respectively (Fig. S2, *A–C*).

### Transmission electron microscopy

For EM, tissue samples were prepared by fixation, embedding, and ultra-microtome sectioning. Paraformaldehyde-fixed tissue was incubated at 4 °C overnight with Karnovsky fixative, containing 2% formaldehyde (Sigma-Aldrich, Stockholm, Sweden), and 2% glutaraldehyde (Agar Scientific Ltd., Stansted, UK) in 0.1 m sodium cacodylate buffer (Agar Scientific). Tissue was washed with 0.1 m sodium cacodylate buffer and postfixed with 2% osmium tetroxide (Agar Scientific) in 0.1 m sodium cacodylate buffer at room temperature in the dark for 2 h. Dehydration was done with rising concentrations of ethanol (50, 70, 95, and 99.5%) and later with 100% acetone and embedded in Agar 100 resin (Agar Scientific). Semi-thin tissue sections were obtained with an ultra-microtome (Leica EM UC6), placed onto copper grids (PELCO GRIDS 200, Ted Pella, Inc.), and post-stained with uranyl acetate and Reynolds lead citrate.

EM observations were carried out on a GAIA3 FIB-SEM work station using a STEM detector (GAIA3; Tescan, Brno-Kohoutovice, Czech Republic) at 30.0 kV (Chalmers Materials Analysis Laboratory, Chalmers University of Technology, Gothenburg, Sweden) (Fig. S2*D*).

### Spectral analysis and laser microdissection

Spectral imaging was performed using an LSM 710 NLO laser-scanning microscope equipped with a 34-channel QUASAR detector (Zeiss). A plan-apochromat ×20/0.8 (WD = 0.55 mm), ∞/0.17 objective was used for spectral imaging of amyloid deposits prior to their isolation. Continuous emission was acquired in the range of 405–750 nm (10, 13). Linear unmixing, a function within the Zen 2011 (Zeiss) software, was used to differentiate between the true q-FTAA and h-FTAA fluorescent signals in the double-stained samples and distinguish between true LCO fluorescence spectrum and unwanted autofluorescence from, for instance, lipofuscin (13). For hyperspectral differentiation based on the hyperspectral line scan, an in-house developed macro for ImageJ (National Institutes of Health) was used. The macro allows the detection of the wavelength showing the normalized intensity for each position (pixel) in the region of interest. Amyloid plaques and CAA were chosen randomly and plaques were subcategorized into cored and diffuse deposits, based on their line-scan profiles. Plaques were annotated based on their LCO profile by three independent investigators. In s-AD tissue, a total of 200–250 cored plaques and 200–250 diffuse plaques and ∼50 CAA deposits were collected from five consecutive, temporal cortical sections. In CU-AP tissue, a total of 200–250 diffuse plaques were collected from five consecutive, temporal cortical sections. This was sufficient for extraction and provided the necessary MS signal. For transgenic mice, a number of 15–20 cored plaques and 15–20 diffuse plaques were each collected from cortex and hippocampus from five consecutive, sagittal sections. Here, the number of plaques was smaller, as the amyloid content in transgenics is significantly higher due to overexpression. In addition, 15–25 CAA deposits were collected per animal from five consecutive sagittal sections per animal.

By investigating plaques at different sections for each patient or animal brain sample, we ensured that only truly diffuse or truly cored plaques were excised. This was to prevent classification of a diffuse corona of a cored plaque as a diffuse plaque, as truly diffuse plaques span over several sections. At the same time, this also provided a representative coverage of biological variation within each brain sample, by including plaques from different sections.

Annotated plaques were then excised by laser microdissection pressure catapulting. Microdissection was done using a PALM Microbeam LMPC microscope (Zeiss) equipped with a 355-nm pulsed UV laser. The spectrally differentiated Aβ plaque subpopulations and CAAs were collected in Adhesive Cap 500 opaque tubes (Zeiss) and stored at −20 °C prior to extraction.

### Aβ immunoprecipitation, Aβ quantification, and MS

To the isolated amyloid aggregates, 50 μl of 70% formic acid with 5 mm EDTA was added, and the samples were sonicated for 5 min and incubated for 1 h at 24 °C. The samples were then neutralized to pH 7 using 0.5 m Tris. Aβ peptides were than purified through immunoprecipitation using Aβ-specific antibodies (antibodies 6E10 and 4G8, Signet Laboratories), coupled to magnetic Dynabeads M-280 sheep anti-mouse (Invitrogen, Carlsbad, CA) as described previously (13, 72). The supernatant was collected and dried through lyophilization. Mass spectrometric comparison of the samples was performed using a MALDI TOF/TOF UltrafleXtreme instrument (Bruker Daltonics, Bremen, Germany) as described previously (13, 72). Further, to verify the identity of the observed peptides, an LC-MS/MS analysis, using the alkaline mobile phase, of brain tissue was carried out using a Q Exactive quadrupole–Orbitrap hybrid mass spectrometer equipped with a heated electrospray ionization source (HESI-II) (Thermo Scientific, Waltham, MA) and UltiMate 3000 binary pump, column oven, and autosampler (Thermo Scientific), as described previously (73), but with the Q Exactive operated in data-dependent mode. Briefly, the resolution settings were 70,000, and target values were 1 × 10^6^ both for MS and MS/MS acquisitions. Acquisitions were performed with 1 microscan/acquisition. Precursor isolation width was 3 *m*/*z* units, and ions were fragmented by so-called higher-energy collision-induced dissociation at a normalized collision energy of 25.

### Data processing and statistical analysis

For statistical analysis, individual mass spectra were exported as csv files from FlexAnalysis (version 3.0, Bruker Daltonics) and imported into Origin (version 8.1 OriginLab, Northampton, MA). Bin borders were used for area under curve (AUC) peak integration within each bin using an in-house developed R script, as described before (74). Individual peptide signal was normalized to all detected and verified peptides. Analysis of individual peptide signals and comparisons between the groups were performed with paired (s-AD, same animal) and unpaired (s-AD/CU-AP and between ages), two-tailed *t* test; correlation between the variables was accessed using Pearson regression analysis. A *p* value threshold of 0.05 was used for assessment of the statistical significance. Statistical analysis was performed using GraphPad Prism (version 7, San Diego, CA). The high resolution orbitrap spectra were deconvoluted using Mascot Distiller before submission to a database search using the Mascot search engine (both from Matrix Science) as described previously (75). The MS/MS spectra were searched against the SwissProt database containing the mutant human APP sequence using the following search parameters: taxonomy: *Homo sapiens*; precursor mass: ±15 ppm; fragment mass: ±0.05 Da; no enzyme; no fixed modifications; variable modifications: deamidation (NQ), Glu → pyro-Glu (N-term E), oxidation (M); instrument: default. For illustration, spectra were processed and searched using PEAKS Studio version 8.5 (Bio-informatics Solutions, Inc., Waterloo, Canada) (Figs. S3–S5).

### Tissue preparation and MALDI imaging MS of Aβ peptides

For MALDI imaging, consecutive tissue sections to those collected for LMPC on PEN membrane slides were thaw-mounted on conductive indium tin oxide glass slides (Bruker Daltonics). A series of sequential washes of 100% EtOH (60 s), 70% EtOH (30 s), Carnoy's fluid (6:3:1 EtOH/chloroform/acetic acid) (110 s), 100% EtOH (15 s), H_2_O with 0.2% TFA (60 s), and 100% EtOH (15 s) was carried out. Tissue was subjected to formic acid vapor for 20 min. 2,5-Dihydroxyacetophenone was used as matrix compound and applied using a TM Sprayer (HTX Technologies, Chapel Hill, NC). A matrix solution of 15 mg/ml 2,5-dihydroxyacetophenone in 70% acetonitrile, 2% acetic acid, 2% TFA was sprayed onto the tissue sections using the following instrumental parameters: nitrogen flow (10 p.s.i.), spray temperature (75 °C), nozzle height (40 mm), eight passes with offsets and rotations, spray velocity (1000 mm/min), and isocratic flow of 100 μl/min using 70% acetonitrile as pushing solvent. Following the matrix deposition, the preparations were recrystallized with 5% methanol at 85 °C for 3 min as described previously (70, 76).

MALDI IMS was performed with 25 μm spatial resolution on a Bruker UltrafleXtreme instrument equipped with a SmartBeam II Nd:YAG/355-nm laser as described previously (70). For verification of Aβ peptide distribution in tissue, image data were reconstructed, normalized to the total ion current, and visualized using Flex Imaging version 3.0 software (Bruker Daltonics).

## Author contributions

J. H. and W. M. conceived and designed the study. S. S. and D. S. provided the mouse brain samples. T. L. selected and provided the human brain samples. W. M., S. N., P. H., and G. B. performed experiments. W. M., P. W., T. L., L. G., G. B., and J. H. analyzed and interpreted the data. W. M., S. N., P. W., T. L., G. B., S. S., D. S., I. K., D. B., L. G., K. P. R. N., P. H., K. B., H. Z., and J. H. discussed the data. W. M., K. B., H. Z., and J. H. wrote the manuscript. All authors approved the final version of the manuscript.

## Supplementary Material

Supporting Information

## Footnotes

3 The abbreviations used are:

AD: Alzheimer's disease
q- and h-FTAA: tetra- and heptameric formyl-thiophene acetic acid, respectively
LCO: luminescent conjugated oligothiophene
CU-AP: cognitively unaffected, amyloid-positive
Aβ: amyloid-β
s-AD: sporadic AD
LMPC: laser microdissection with pressure catapulting
IP: immunoprecipitation
CR: Congo red
pE: pyroglutamate
IMS: imaging mass spectrometry
CAA: cerebral amyloid angiopathy
CSF: cerebrospinal fluid
APP: amyloid precursor protein
AUC: area under curve.
