# Supplementary material for: Pyroglutamation of amyloid-βx-42 (Aβx-42) followed by Aβ1–40 deposition underlies plaque polymorphism in progressing Alzheimer's disease pathology
Source: J Biol Chem. 2019 Feb 27;294(17):6719–32. doi: 10.1074/jbc.RA118.006604 (PMC6497931; doi:10.1074/jbc.RA118.006604)
Supplement: Supporting Information [file supp_294_17_6719__index.html]

Pyroglutamation of amyloid-βx-42 (Aβx-42) followed by Aβ1–40 deposition underlies plaque polymorphism in progressing Alzheimer's disease pathology — Molecular evolution of amyloid plaque polymorphism — Supporting Information 

# Pyroglutamation of amyloid-βx-42 (Aβx-42) followed by Aβ1–40 deposition underlies plaque polymorphism in progressing Alzheimer's disease pathology

## Supporting Information

- Supporting Information (to be published online) - Figure S1-S7, Table S1
