# Supplementary material for: Pyroglutamation of amyloid-βx-42 (Aβx-42) followed by Aβ1–40 deposition underlies plaque polymorphism in progressing Alzheimer's disease pathology
Source: J Biol Chem. 2019 Feb 27;294(17):6719–32. doi: 10.1074/jbc.RA118.006604 (PMC6497931; doi:10.1074/jbc.RA118.006604)
Supplement: Supporting Information [file supp_RA118.006604_141802_2_supp_292561_pnlcrf.pdf]

Supporting Information

Pyroglutamation of amyloid-βx-42 (Aβx-42) followed by Aβ1-40 deposition underlies plaque polymorphism in progressing Alzheimer's disease pathology

Wojciech Michno[1], Sofie Nyström[2], Patrick Wehrli[1], Tammaryn Lashley[3], Gunnar Brinkmalm[1], Laurent Guerard[1], Stina Syvänen[4], Dag Sehlin[4], Ibrahim Kaya[1], Dimitri Brinet[1], K. Peter R. Nilsson[2], Per Hammarström[2], Kaj Blennow[1,5], Henrik Zetterberg[1,3,5,6], and Jörg Hanrieder[1,3,6]*

From the [1]Department of Psychiatry and Neurochemistry, Sahlgrenska Academy at the University of Gothenburg, Mölndal, Sweden; [2]Department of Physics, Chemistry and Biology, Linköping University, Linköping, Sweden; [3]Department of Neurodegenerative Disease, UCL Queen Square Institute of Neurology, University College London, London, United Kingdom; [4]Department of Public Health and Caring Sciences, Uppsala University, Uppsala, Sweden; [5]Clinical Neurochemistry Laboratory, Sahlgrenska University Hospital, Mölndal, Sweden; [6]UK Dementia Research Institute at UCL, London, United Kingdom

Content:

Table S1

Figures S1-S7

Table S1. Patient characteristics of subjects included in the study.

Case	Etiology	Age at onset, years	Age at death, years	Sex	Braak staging	Thal staging	CERAD score	ABC Score
AD1	sAD	51	62	F	6	5	3:3	A3B3C3
AD2	sAD	69	81	M	6	5	3:3	A3B3C3
AD3	sAD	58	68	M	6	5	3:3	A3B3C3
AD4	sAD	62	72	M	6	5	3:3	A3B3C3
AD5	sAD	70	86	F	6	5	3:3	A3B3C3
AD6	sAD	55	64	M	6	5	3:3	A3B3C3
AD7	sAD	68	80	M	6	5	3:3	A3B3C3
AD8	sAD	50	66	F	6	5	3:3	A3B3C3
CU-AP1	CU-AP		88	M	3	5	2:3	A3B2C2
CU-AP2	CU-AP		95	F	4	4	2:2	A3B2C2
CU-AP3	CU-AP		87	F	4	3	2:3	A2B2C2
CU-AP4	CU-AP		91	F	4	1	3:3	A3B2C2

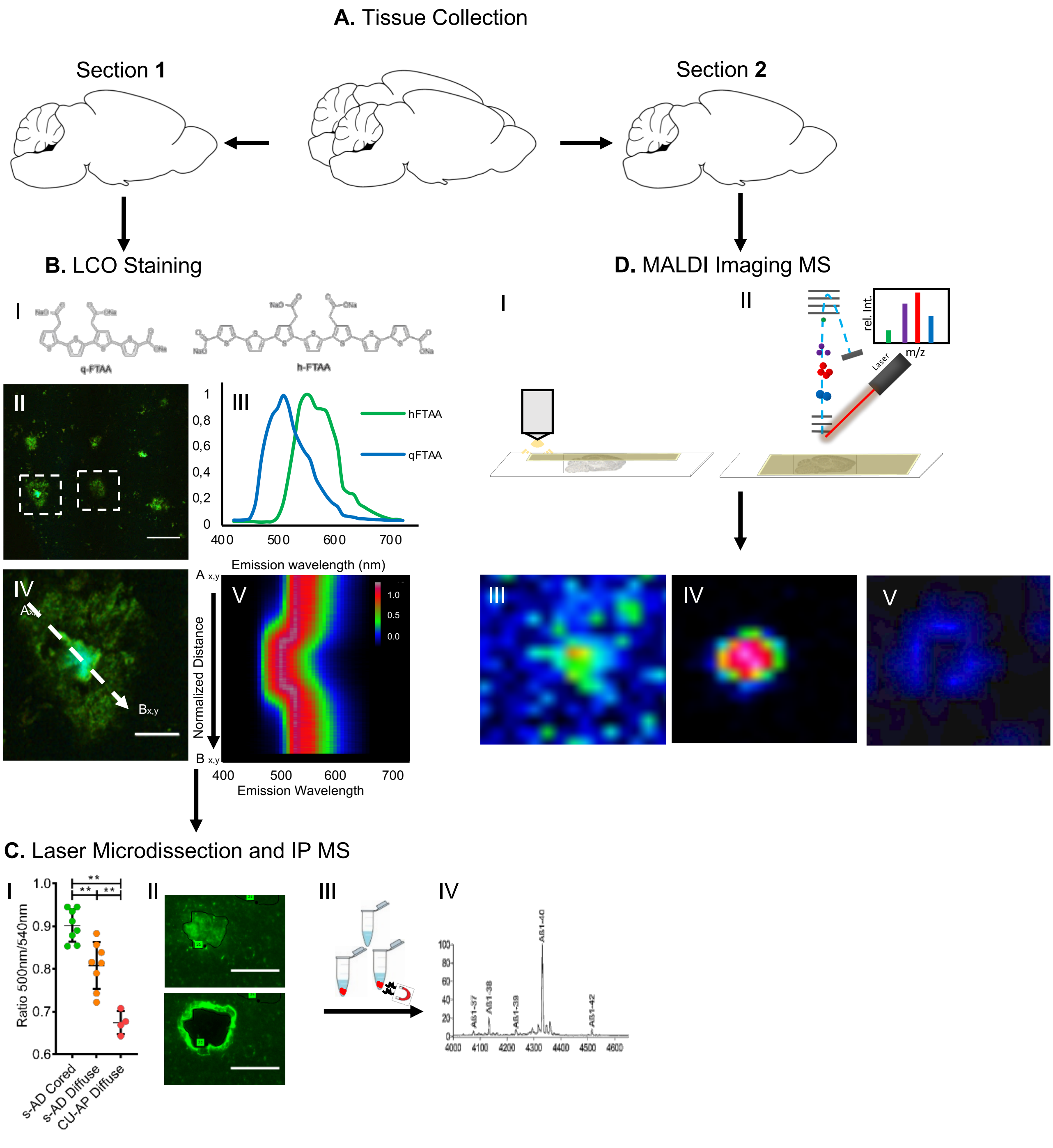

Figure S1. Experimental Setup. Multimodal Chemical Imaging paradigm for analysis of tgAPP$_{SWE}$ and s-AD/CU-AP brain tissue. (A) Cryo-sectioning and collection of 12um brains sections that were thaw-mounted onto LMD membrane slides for laser microdissection (Section I) and onto conductive, indium tin oxide coated glass slides for MALDI imaging mass spectrometry (Section 2). (B.I) Section 1 was used for double staining with q- and h-FTAA. (B.II) Fluorescence imaging of LCO stained plaque pathology followed by (B.III) LCO spectral acquisition and (B.IV,V) line scan analysis across polymorphic plaques. Here, (B.V) a blue shift indicates q-FTAA binding as observed for the core region of neuritic plaques in s-AD. (C.I) Plaques morphotypes could be classified based on their hyperspectral profile and gave characteristic emission ratio values (500nm/540nm) corresponding to the degree of q- and h-FTAA content. (C.II) Hyperspectrally delineated plaques were excised by laser microdissection. (C.III) Plaques were extracted with formic acid followed by Aβ enrichment with Immunoprecipitation IP. (C.IV) Individual Aβ species were identified and relatively quantified with MALDI mass spectrometry (see also Figure 1B-D).

(D) MALDI IMS was performed on consecutive sections (Section 2) to the sections used for LCO imaging and LMPC. Here, (D.I) the tissue section was coated with crystalline, small organic matrix. (D.II) This was followed by laser irradiation based desorption and ionization (MALDI) and ion detection with a time of flight mass analyzer generating MALDI TOF MS data from distinct coordinates of the tissue section. Acquisition of MALDI MS in a pre-defined pattern can generate distinct ion distribution map (single ion images). This is exemplified for Aβ peptides in human s-AD tissue with Aβ 1-42 localizing to diffuse plaques (D.III) and Aβ 1-40 localizing to the center of cored plaques (D.IV) as well as to CAA (D.V).

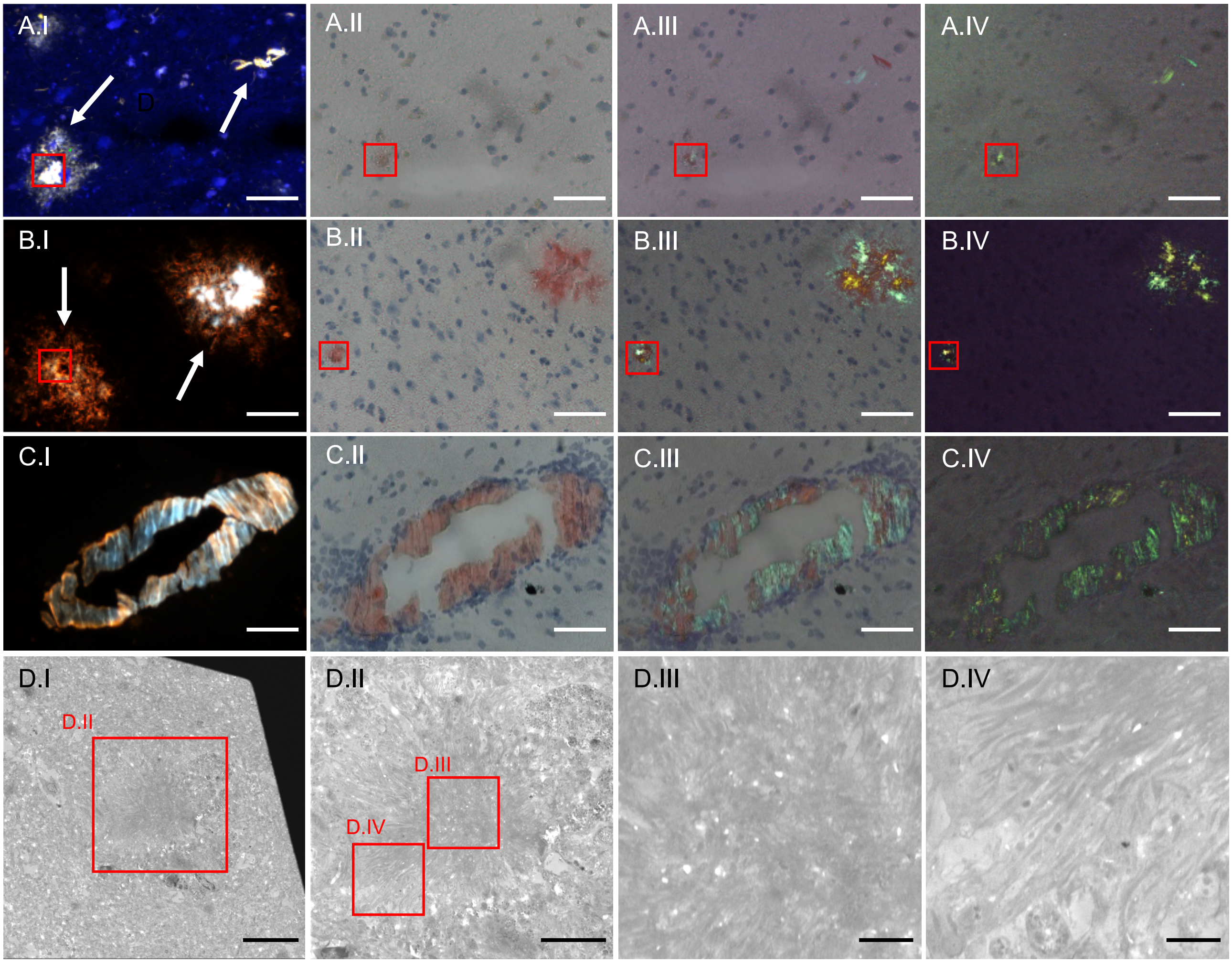

Figure S2. Double staining of LCO with Congo red (CR) and Electronmicroscopy in human and mouse Aβ plaque pathology.

(A) Complementary LCO/CR staining in human s-AD brain tissue using hFTAA/qFTAA double staining of plaques and Congo red in adjacent sections. (A.I) In detail, a cored plaque (left arrow), with a blue-shifted center (red box), and neurofibrillary tau tangle (NFT) (right arrow) in human s-AD patient, reveal a pattern of qFTAA/hFTAA blue-shift correspondence to CR birefringence. This is clearly seen in the (A.II) non-polarized, (A.III) semi-polarized, and polarized light images of CR (A.IV). Here birefringence is present only in the blue-shifted plaque center (red square) (A.IV) .

(B—C) LCO Double staining with hFTAA and qFTAA and Congo red staining in adjacent sections in 18-month old $tgAPP_{SWE}$ mice. (B.I) Largely diffuse plaque (left arrow) with a light blue-shift in the center (red box) and the blue-shifted cored plaque (right arrow) in $tgAPP_{SWE}$ are shown with adjacent sections stained with CR as seen in (B.II) non-polarized, (B.III) semi-polarized, and (B.IV) polarized light, indicate, similarly to human data, a clear presence of birefringence in the blue-shifted cored plaque (right arrow), and a minor birefringence in the lightly blue-shifted portion of the diffuse deposit. (C.I) A mainly blue-shifted cerebral amyloid angiopathy (CAA) in $tgAPP_{SWE}$, display a clear birefringence in CR staining as seen in (C.II) non-polarized, (C.III) semi-polarized, and (C.IV) polarized light.

(D.I) Electromicrogram of cored Aβ plaque, from $tgAPP_{SWE}$ mouse (red square), (D.II) zoom, revealed clear structural diversity across the Aβ deposit (red squares). This is clearly seen in (D.III) distorted, densely core, as compared to the (D.IV) organized fibrillary structures in the periphery of the Aβ plaque. Scale bar: (A-C) 50µm; (D.I) 20µm, (D.II) 10µm, (D.III,D.IV) 2µm.

A

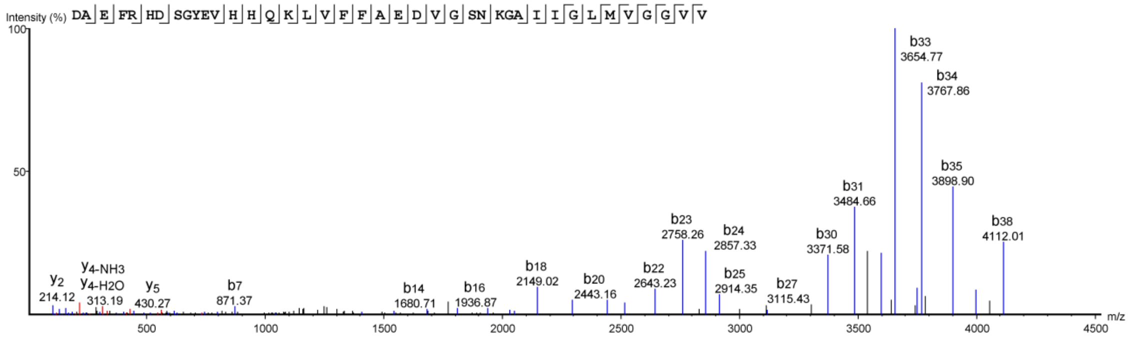

B

#	Immonium	b	b-H2O	b-NH3	Seq	y	y-H2O	y-NH3	#
1	88.04	116.03	98.02	99.01	D				40
2	44.05	187.07	169.10	170.04	A	4213.13	4195.12	4196.10	39
3	102.06	316.11	298.10	299.09	E	4142.09	4124.08	4125.06	38
4	120.08	463.18	445.17	446.16	F	4013.05	3995.04	3996.02	37
5	129.10	619.28	601.27	602.26	R	3865.98	3847.97	3848.95	36
6	110.07	756.34	738.33	739.32	H	3709.88	3691.87	3692.85	35
7	88.04	871.37	853.36	854.34	D	3572.82	3554.81	3555.79	34
8	60.04	958.40	940.39	941.37	S	3457.79	3439.78	3440.77	33
9	30.03	1015.42	997.41	998.40	G	3370.76	3352.75	3353.73	32
10	136.08	1178.49	1160.48	1161.46	Y	3313.74	3295.73	3296.71	31
11	102.06	1307.53	1289.52	1290.50	E	3150.68	3132.67	3133.65	30
12	72.08	1406.59	1388.59	1389.57	V	3021.63	3003.62	3004.61	29
13	110.07	1543.65	1525.65	1526.63	H	2922.57	2904.56	2905.54	28
14	110.07	1680.71	1662.70	1663.69	H	2785.51	2767.50	2768.48	27
15	101.07	1808.77	1790.76	1791.75	Q	2648.45	2630.44	2631.42	26
16	101.11	1936.87	1918.86	1919.84	K	2520.39	2502.38	2503.36	25
17	86.10	2049.95	2031.94	2032.93	L	2392.29	2374.28	2375.27	24
18	72.08	2149.02	2131.01	2131.99	V	2279.21	2261.20	2262.18	23
19	120.08	2296.08	2278.08	2279.06	F	2180.14	2162.13	2163.11	22
20	120.08	2443.16	2425.15	2426.13	F	2033.07	2015.06	2016.05	21
21	44.05	2514.19	2496.18	2497.17	A	1886.01	1867.99	1868.98	20
22	102.06	2643.23	2625.23	2626.21	E	1814.97	1796.96	1797.94	19
23	88.04	2758.26	2740.25	2741.24	D	1685.93	1667.91	1668.90	18
24	72.08	2857.33	2839.32	2840.31	V	1570.90	1552.89	1553.87	17
25	30.03	2914.35	2896.34	2897.33	G	1471.83	1453.82	1454.80	16
26	60.04	3001.39	2983.38	2984.36	S	1414.81	1396.80	1397.78	15
27	87.06	3115.43	3097.42	3098.40	N	1327.78	1309.77	1310.75	14
28	101.11	3243.52	3225.51	3226.50	K	1213.73	1195.72	1196.71	13
29	30.03	3300.55	3282.54	3283.52	G	1085.64	1067.63	1068.61	12
30	44.05	3371.58	3353.57	3354.56	A	1028.62	1010.61	1011.59	11
31	86.10	3484.66	3466.66	3467.64	I	957.58	939.57	940.55	10
32	86.10	3597.75	3579.74	3580.72	I	844.50	826.49	827.47	9
33	30.03	3654.77	3636.76	3637.75	G	731.41	713.40	714.39	8
34	86.10	3767.86	3749.85	3750.84	L	674.39	656.38	657.36	7
35	104.05	3898.90	3880.89	3881.87	M	561.31	543.30	544.28	6
36	72.08	3997.97	3979.96	3980.94	V	430.27	412.25	413.26	5
37	30.03	4054.99	4036.98	4037.96	G	331.20	313.19	314.19	4
38	30.03	4112.01	4094.00	4094.98	G	274.18	256.17	257.13	3
39	72.08	4211.08	4193.07	4194.05	V	217.15	199.18	200.13	2
40	72.08				V	118.09	100.08	101.06	1

Figure S3. LC-MS/MS based verification of Aβ1-40. (A) MS/MS fragment mass spectrum of Aβ1-40, where cleavage sites are indicated in the peptide sequence. (B) Table of detected and matched fragment ions indicating sequence coverage for Aβ1-40. The detected and accurately matched MS/MS fragment ions are color indicated in the spectrum and table (b-ions and amino acid immonium ions blue; y-ions: red)

A

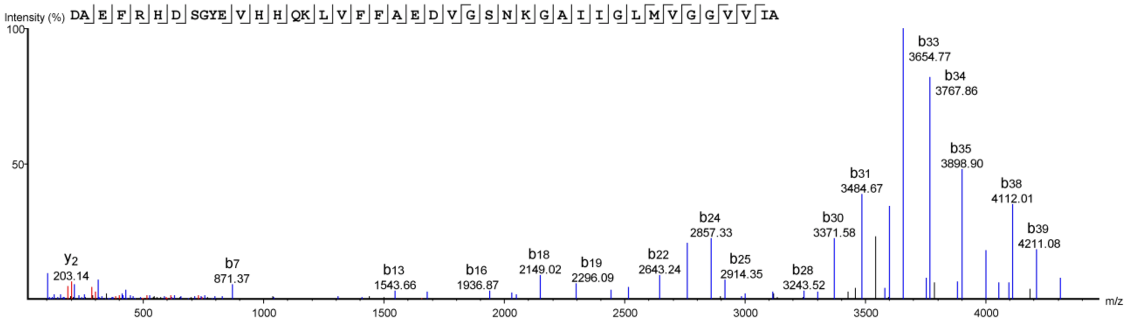

B

#	Immonium	b	b-H2O	b-NH3	Seq	y	y-H2O	y-NH3	#
1	88.04	116.03	98.02	99.01	D				42
2	44.05	187.07	169.10	170.04	A	4397.25	4379.24	4380.22	41
3	102.06	316.11	298.10	299.09	E	4326.21	4308.20	4309.19	40
4	120.08	463.18	445.17	446.16	F	4197.17	4179.16	4180.14	39
5	129.10	619.28	601.27	602.26	R	4050.10	4032.09	4033.07	38
6	110.07	756.34	738.33	739.32	H	3894.00	3875.99	3876.97	37
7	88.04	871.37	853.36	854.34	D	3756.94	3738.93	3739.91	36
8	60.04	958.40	940.39	941.37	S	3641.91	3623.90	3624.89	35
9	30.03	1015.42	997.41	998.40	G	3554.88	3536.87	3537.86	34
10	136.08	1178.49	1160.48	1161.46	Y	3497.86	3479.85	3480.83	33
11	102.06	1307.53	1289.52	1290.50	E	3334.80	3316.79	3317.77	32
12	72.08	1406.60	1388.59	1389.57	V	3205.76	3187.74	3188.73	31
13	110.07	1543.66	1525.65	1526.63	H	3106.69	3088.68	3089.66	30
14	110.07	1680.71	1662.70	1663.69	H	2969.63	2951.62	2952.60	29
15	101.07	1808.77	1790.76	1791.75	Q	2832.57	2814.56	2815.54	28
16	101.11	1936.87	1918.86	1919.84	K	2704.51	2686.50	2687.48	27
17	86.10	2049.95	2031.94	2032.93	L	2576.42	2558.41	2559.39	26
18	72.08	2149.02	2131.01	2131.99	V	2463.33	2445.32	2446.30	25
19	120.08	2296.09	2278.08	2279.06	F	2364.26	2346.25	2347.24	24
20	120.08	2443.15	2425.15	2426.13	F	2217.19	2199.18	2200.17	23
21	44.05	2514.19	2496.18	2497.17	A	2070.13	2052.12	2053.10	22
22	102.06	2643.24	2625.23	2626.21	E	1999.09	1981.08	1982.06	21
23	88.04	2758.27	2740.25	2741.24	D	1870.05	1852.04	1853.02	20
24	72.08	2857.33	2839.32	2840.31	V	1755.02	1737.01	1737.99	19
25	30.03	2914.35	2896.34	2897.33	G	1655.95	1637.94	1638.92	18
26	60.04	3001.38	2983.36	2984.36	S	1598.93	1580.92	1581.90	17
27	87.06	3115.43	3097.42	3098.40	N	1511.90	1493.89	1494.87	16
28	101.11	3243.52	3225.51	3226.50	K	1397.85	1379.84	1380.83	15
29	30.03	3300.54	3282.54	3283.52	G	1269.76	1251.75	1252.73	14
30	44.05	3371.58	3353.57	3354.56	A	1212.74	1194.73	1195.71	13
31	86.10	3484.67	3466.66	3467.64	I	1141.70	1123.69	1124.67	12
32	86.10	3597.75	3579.74	3580.73	I	1028.62	1010.61	1011.59	11
33	30.03	3654.77	3636.76	3637.75	G	915.53	897.52	898.51	10
34	86.10	3767.86	3749.85	3750.84	L	858.51	840.50	841.48	9
35	104.05	3898.90	3880.89	3881.88	M	745.43	727.41	728.40	8
36	72.08	3997.96	3979.96	3980.94	V	614.39	596.38	597.37	7
37	30.03	4054.99	4036.98	4037.96	G	515.32	497.34	498.27	6
38	30.03	4112.01	4094.00	4095.00	G	458.30	440.29	441.27	5
39	72.08	4211.08	4193.07	4194.05	V	401.28	383.27	384.26	4
40	72.08	4310.14	4292.13	4293.12	V	302.21	284.20	285.19	3
41	86.10	4423.23	4405.22	4406.20	I	203.14	185.16	186.12	2
42	44.05				A	90.05	72.04	73.03	1

Figure S4. LC-MS/MS based verification of Aβ1-42. (A) MS/MS fragment mass spectrum of Aβ1-42, where cleavage sites are indicated in the peptide sequence. (B) Table of detected and matched fragment ions indicating sequence coverage for Aβ1-42. The detected and accurately matched MS/MS fragment ions are color indicated in the spectrum and table (b-ions and amino acid immonium ions blue; y-ions: red)

A

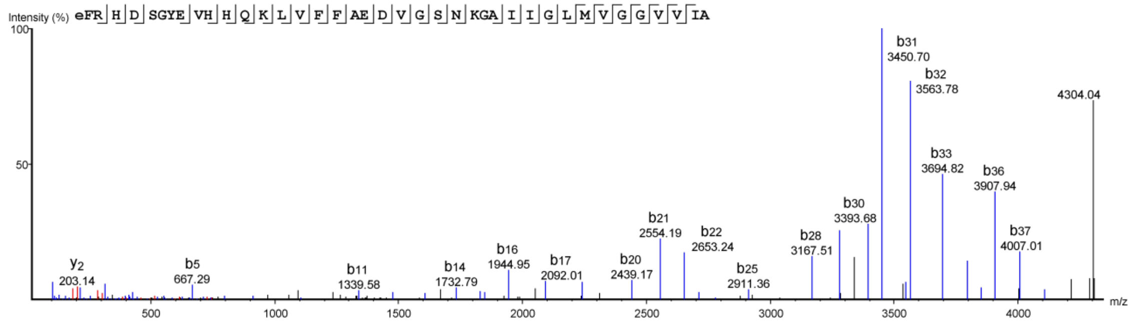

B

#	Immonium	b	b-H2O	b-NH3	Seq	y	y-H2O	y-NH3	#
1	84.04	112.04	94.03	95.01	E(-18.01)				40
2	120.08	259.11	241.10	242.08	F	4197.17	4179.16	4180.14	39
3	129.10	415.21	397.20	398.18	R	4050.10	4032.09	4033.07	38
4	110.07	552.27	534.26	535.24	H	3894.00	3875.99	3876.97	37
5	88.04	667.29	649.28	650.27	D	3756.94	3738.93	3739.91	36
6	60.04	754.33	736.32	737.30	S	3641.91	3623.90	3624.89	35
7	30.03	811.35	793.34	794.32	G	3554.88	3536.87	3537.86	34
8	136.08	974.41	956.40	957.39	Y	3497.86	3479.85	3480.83	33
9	102.06	1103.45	1085.44	1086.43	E	3334.80	3316.79	3317.77	32
10	72.08	1202.52	1184.51	1185.50	V	3205.76	3187.74	3188.73	31
11	110.07	1339.58	1321.57	1322.55	H	3106.69	3088.68	3089.66	30
12	110.07	1476.64	1458.63	1459.61	H	2969.63	2951.62	2952.60	29
13	101.07	1604.70	1586.69	1587.67	Q	2832.57	2814.56	2815.54	28
14	101.11	1732.79	1714.78	1715.77	K	2704.51	2686.50	2687.48	27
15	86.10	1845.88	1827.87	1828.85	L	2576.42	2558.41	2559.39	26
16	72.08	1944.95	1926.94	1927.92	V	2463.33	2445.32	2446.30	25
17	120.08	2092.01	2074.00	2074.99	F	2364.26	2346.25	2347.24	24
18	120.08	2239.09	2221.07	2222.06	F	2217.19	2199.18	2200.17	23
19	44.05	2310.12	2292.11	2293.09	A	2070.13	2052.12	2053.10	22
20	102.06	2439.17	2421.15	2422.14	E	1999.09	1981.08	1982.06	21
21	88.04	2554.19	2536.18	2537.16	D	1870.05	1852.04	1853.02	20
22	72.08	2653.24	2635.25	2636.23	V	1755.02	1737.01	1737.99	19
23	30.03	2710.27	2692.27	2693.25	G	1655.95	1637.94	1638.92	18
24	60.04	2797.31	2779.30	2780.29	S	1598.93	1580.92	1581.90	17
25	87.06	2911.36	2893.34	2894.33	N	1511.90	1493.89	1494.87	16
26	101.11	3039.45	3021.44	3022.42	K	1397.85	1379.84	1380.83	15
27	30.03	3096.47	3078.46	3079.44	G	1269.76	1251.75	1252.73	14
28	44.05	3167.51	3149.50	3150.48	A	1212.74	1194.73	1195.71	13
29	86.10	3280.60	3262.58	3263.57	I	1141.70	1123.69	1124.67	12
30	86.10	3393.68	3375.67	3376.65	I	1028.62	1010.61	1011.59	11
31	30.03	3450.70	3432.69	3433.67	G	915.53	897.52	898.51	10
32	86.10	3563.78	3545.77	3546.76	L	858.51	840.50	841.48	9
33	104.05	3694.82	3676.81	3677.80	M	745.43	727.41	728.40	8
34	72.08	3793.88	3775.88	3776.86	V	614.39	596.38	597.37	7
35	30.03	3850.92	3832.90	3833.89	G	515.32	497.34	498.27	6
36	30.03	3907.94	3889.92	3890.91	G	458.30	440.29	441.27	5
37	72.08	4007.01	3988.99	3989.98	V	401.28	383.27	384.26	4
38	72.08	4106.07	4088.06	4089.04	V	302.21	284.20	285.19	3
39	86.10	4219.15	4201.14	4202.13	I	203.14	185.16	186.12	2
40	44.05				A	90.05	72.04	73.03	1

Figure S5. LC-MS/MS based verification of Aβ3pE-42. (A) MS/MS fragment mass spectrum of Aβ3pE-42, where cleavage sites are indicated in the peptide sequence. (B) Table of detected and matched fragment ions indicating sequence coverage for Aβ3pE-42. The detected and accurately matched MS/MS fragment ions are color indicated in the spectrum and table (b-ions and amino acid immonium ions blue; y-ions: red)

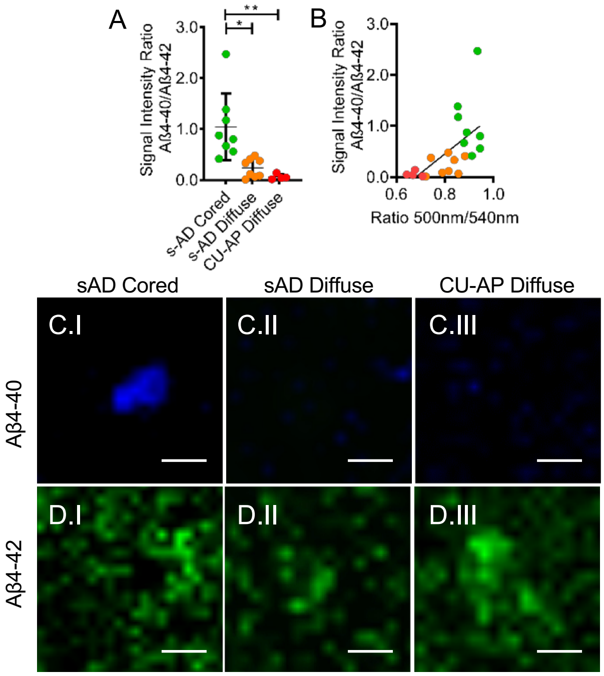

Figure S6. Quantification and localization of N-terminally truncated Aβ4-x peptides in diffuse and cored plaques. (A) Signal ratio in between plaque types in AD and CU-AP. (B) correlation with 500nm/540nm emission ratio for Aβ4-40/Aβ4-42 (R^2=0.3650, $p<0.01$). Single ion images of the N-terminally truncated (C.I-III) Aβ4-40 and (D.I-III) Aβ4-42, revealed similar localization patterns as for the non-truncated forms of these peptides (i.e. Aβ1-40 and Aβ1-42, see figure 1G). This in support of the observation that the Aβ1-40 peptide is characteristic of the core formation. Nr. of patients n=8 (s-AD), n=4 (CU-AP); A number of 200-250 cored plaques, and 200-250 diffuse plaques for s-AD, and 200-250 diffuse plaques for CU-AP, were collected from 5 consecutive, temporal cortical sections per patient; MALDI IMS was performed on consecutive sections to the sections used for LCO imaging and LMPC.
Errorbars (A): S.D; Scale bar: (C-D) 20µm; Significance: *$p<0.05$; **$p<0.005$.

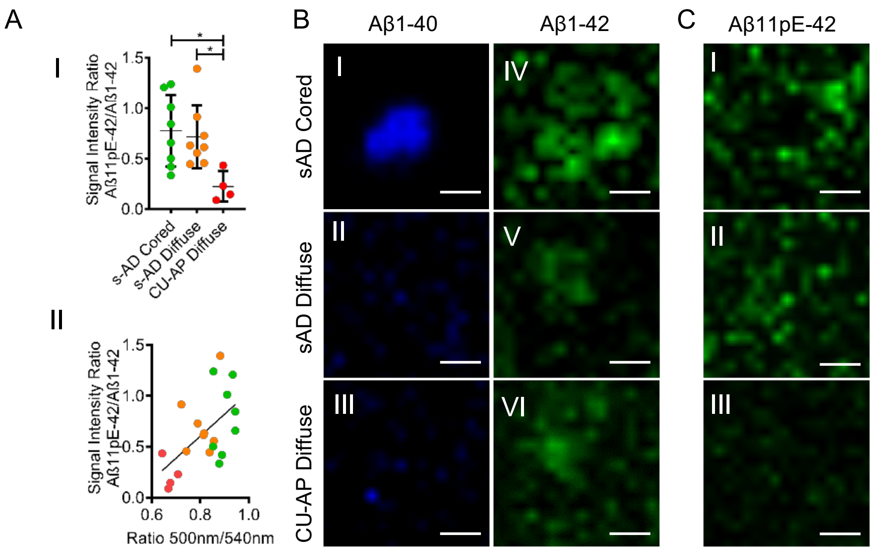

Figure S7. Quantification and localization of N-terminally truncated Aβ11pE-x peptides in diffuse and cored plaques. (A.I) Signal ratio in between plaque types in AD and CU-AP. (A.II) Correlation with 500nm/540nm emission ratio for Aβ 11pE-42/Aβ1-42 (R^2=0.3189, p<0.01).

IMS analysis revealed a prominent localization of Aβ1-40 to the center of the cored plaques in s-AD (B.I), while the Aβ1-42 signal localized to the periphery of cored plaques in s-AD tissue. Diffuse plaques in both s-AD (B.II, B.V) and CU-AP (B.III, B.VI) showed low Aβ1-40 signal, but a strong Aβ1-42 signal, that was homogenous across these plaques. MALDI IMS analysis did further reveal localization of AβpE11-42 to the periphery of cored plaques in s-AD (C.I) as well as diffuse plaques in s-AD (C.II), while only a very low signal was present for these peptides in the diffuse plaques in CU-AP (C.III). Nr. of patients n=8 (s-AD), n=4 (CU-AP); A number of 200-250 cored plaques, and 200-250 diffuse plaques for s-AD, and 200-250 diffuse plaques for CU-AP, were collected from 5 consecutive, temporal cortical sections per patient; MALDI IMS was performed on consecutive sections to the sections used for LCO imaging and LMPC. Errorbars (A): S.D.; Scale bar: (C) 20µm; Significance: *p<0.05.
